# *Nanog*^+^F10-Derived Extracellular Vesicles Suppress Melanoma Metastasis, Implicating *miR-19a-3p* in Macrophage-Dependent Innate Immune Regulation

**DOI:** 10.3390/cancers18142200

**Published:** 2026-07-08

**Authors:** Misato Nakano, Asuka Tamura, Sora Yorikawa, Nahoko Matsuki, Runa Ito, Hideaki Matsuoka, Mikako Saito

**Affiliations:** 1 Department of Biotechnology and Life Science, Tokyo University of Agriculture and Technology, 2-24-16, Naka-cho, Koganei, Tokyo 184-8588, Japan; 2 Bioresource Laboratories, Tokyo University of Agriculture and Technology, 2-24-16, Naka-cho, Koganei, Tokyo 184-8588, Japan

**Keywords:** extracellular vesicles, *miR-19a-3p*, melanoma metastasis, macrophages, innate immunity, preemptive immune education, immune modulation, tumor metastasis

## Abstract

Background/Objectives: Extracellular vesicles (EVs) participate in tumor progression and immune regulation through the transfer of bioactive molecules. Although tumor-derived EVs are generally considered to promote metastasis, accumulating evidence suggests that their functions vary according to the biological characteristics of their cells of origin. This study investigated the mechanisms underlying the anti-metastatic effects of EVs derived from *Nanog*-overexpressing melanoma cells (*Nanog*^+^F10-EVs). Methods: *Nanog*^+^F10-EVs were isolated and characterized by EV marker expression. Their anti-metastatic activity was evaluated using melanoma liver metastasis models in Rag2-deficient and macrophage-depleted mice. Differential miRNA expression analysis was performed to identify candidate functional mediators. Functional studies were conducted using *miR-19a-3p*-overexpressing cells and EVs. Results: *Nanog*^+^F10-EVs significantly suppressed melanoma metastasis. Rag2 deficiency only modestly affected this activity, whereas macrophage depletion abolished the anti-metastatic effect, suggesting a predominant role of innate immunity. miRNA profiling identified *miR-19a-3p* as a candidate mediator enriched in *Nanog*^+^F10-EVs. Despite promoting tumor cell proliferation and migration, *miR-19a-3p*-enriched EVs partially reproduced anti-metastatic activity. Macrophage marker analysis suggested that these effects were not fully explained by classical M1/M2 polarization. Conclusions: *Nanog*^+^F10-EVs may suppress metastasis through macrophage-dependent innate immune regulation potentially mediated by *miR-19a-3p* and are consistent with a model of preemptive immune education.

## Simple Summary

Metastasis remains the primary cause of cancer-related mortality, emphasizing the need for novel therapeutic strategies. Extracellular vesicles (EVs) contribute substantially to tumor progression and immune regulation through the transfer of bioactive molecules, including microRNAs. In this study, we examined the anti-metastatic properties of EVs derived from *Nanog*-overexpressing melanoma cells (*Nanog*^+^F10-EVs). Our findings demonstrated that these EVs suppress melanoma metastasis predominantly through macrophage-associated innate immune responses, while adaptive immunity appeared to make a limited contribution. We identified *miR-19a-3p* as a candidate functional mediator highly enriched in *Nanog*^+^F10-EVs. Interestingly, although *miR-19a-3p* enhanced tumor cell proliferation and migration, EVs-mediated delivery of this microRNA induced anti-metastatic effects through immune modulation. These findings raise the possibility that EVs may function not only as carriers of molecular cargo but also may contribute to preemptive immune education that prepare immune cells before the onset of metastatic progression.

## 1. Introduction

Cancer metastasis remains a leading cause of cancer-related mortality, emphasizing the need for the development of novel strategies to control its progression [1]. Increasing evidence has demonstrated that immune cells within the tumor microenvironment, particularly macrophages, play critical roles in the regulation of metastasis [2,3]. Macrophages exhibit substantial plasticity and can adopt diverse functional states, including inflammatory and anti-tumor M1-like phenotypes as well as immunosuppressive and tumor-promoting M2-like phenotypes [2,3]. Therefore, elucidating the molecular mechanisms that govern macrophage functional states is essential for developing strategies aimed at suppressing metastasis.

EVs function as mediators of intercellular communication through the transfer of miRNAs, proteins, lipids, and other bioactive molecules and are closely involved in tumor progression and immune regulation [4,5,6]. Cancer cell-derived EVs are generally recognized for their roles in promoting immune suppression and pre-metastatic niche formation [6,7,8]. However, recent findings suggest that tumor-derived EVs may also alter macrophage function and immune responses under specific conditions [9,10,11]. For example, tumor-derived EVs have been reported to induce macrophages with pro-inflammatory characteristics associated with clinical outcomes [9]. In addition, EVs can stimulate inflammatory cytokine expression in macrophages [10], and they may promote both M1-like and M2-like polarization states [11]. These observations indicate that EVs can exert both immune-suppressive and immune-activating effects, with their biological functions strongly influenced by the characteristics of the cells from which they originate and the molecular cargo they contain [4,5]. Furthermore, even EVs derived from identical tumor cell populations may exhibit distinct functions and differential effects on the tumor microenvironment depending on variations in cellular differentiation status and gene expression profiles [12,13]. Nevertheless, the molecular mechanisms responsible for these functional differences remain largely unclear.

We previously focused on tumor cells exhibiting high expression of the stemness-associated transcription factor Nanog and demonstrated that extracellular vesicles derived from these cells (*Nanog*^+^F10-EVs) suppress cancer metastasis in vivo [14,15,16]. Although cancer cell-derived EVs are widely regarded as promoters of metastasis and pre-metastatic niche formation [6,7,8], these findings suggest that EV functions may vary considerably according to cellular differentiation states and transcriptional programs. Within tumor tissues, cancer stem cells and differentiated tumor cells coexist and secrete EVs with distinct molecular compositions and biological functions, thereby exerting differential effects on metastatic niche formation and tumor progression [12,13]. Thus, EVs may function not merely as tumor-promoting factors but also as immune regulatory mediators that reflect the biological state of their cells of origin. However, the immunological basis underlying the anti-metastatic effects of *Nanog*^+^F10-EVs, particularly the relative contributions of adaptive and innate immunity and their associated molecular mechanisms, remains to be clarified.

Based on these observations, the present study focused on the anti-metastatic effects of *Nanog*^+^F10 cell-derived EVs (*Nanog*^+^F10-EVs) and aimed to elucidate their immunological and molecular mechanisms. Specifically, we investigated the differential contributions of adaptive and innate immunity to metastasis suppression and examined the mechanisms through which EVs regulate immune responses. Furthermore, given the possibility that miRNAs contained within EVs regulate immune cell functions, we explored the mechanisms of metastasis suppression mediated through alterations in macrophage function. miRNA profiling identified *miR-19a-3p* as highly enriched in *Nanog*^+^F10-EVs; therefore, this miRNA was selected for further functional analysis. We investigated whether EVs containing *miR-19a-3p* contribute to metastasis suppression through modulation of innate immune cell functional states. Collectively, this study provides a new perspective on the concept of preemptive immune education, in which EVs may regulate future immune responsiveness before metastatic progression occurs.

## 2. Materials and Methods

### 2.1. Cell Lines and Culture Conditions

The mouse melanoma cell line B16-F10 (F10) and *Nanog*-overexpressing F10 (*Nanog*^+^F10) cells were cultured as previously described [14]. F10 cells overexpressing *miR-19a-3p* (*miR-19a-3p*^+^F10) were generated by transfection with a *miR-19a-3p* expression vector and maintained under the same culture conditions as the other melanoma cell lines. The mouse macrophage cell line J774.1 was obtained from the RIKEN BRC (Tsukuba, Japan) and cultured under identical conditions.

### 2.2. Animals

All animal experiments were conducted in accordance with the Guidelines for the Care and Use of Laboratory Animals of Tokyo University of Agriculture and Technology and were approved by the Institutional Animal Care and Use Committee (IACUC No. No. R06-45, No. R06-49, and R07-57). Male C57BL/6 mice were purchased from Kiwa Laboratory Animals Co., Ltd. (Wakayama, Japan), and Rag2-KO mice were obtained from RIKEN BRC. Rag2 deficiency results in the absence of mature T and B lymphocytes due to impaired V(D)J recombination [17,18]. All animals were maintained under SPF conditions with a 12 h light/dark cycle and allowed to acclimate for at least one week before experimental procedures. C57BL/6 mice aged 8–9 weeks and Rag2-KO mice aged 5–7 weeks were used. Animals were allocated to experimental groups without randomization, and investigators were not blinded during treatment administration or outcome assessment. These represent limitations of the present study.

### 2.3. Preparation of EVs

EVs were isolated using an ultracentrifugation protocol described previously [19]. Protein concentrations were determined using a BCA protein assay following lysis with RIPA buffer. EV purity was assessed by Western blot analysis through detection of EV markers (TSG101, Alix and HSC70) and confirmation of the absence of the Golgi marker GM130 in accordance with MISEV2023 recommendations.

### 2.4. Western Blot Analysis

Cellular protein samples were prepared by lysing cells in RIPA buffer, followed by sonication on ice and centrifugation at 20,000× *g* for 15 min at 4 °C. The resulting supernatants were collected as protein samples. EV pellets obtained following ultracentrifugation were resuspended in RIPA buffer. Protein concentrations were measured using the BCA assay. Proteins were separated by SDS-PAGE and transferred onto PVDF membranes at 100 V for 3 h at 4 °C. Membranes were blocked with 5% (*w*/*v*) skim milk in TBS-T and incubated with primary antibodies diluted in the same buffer for 3 h. Primary antibodies included anti-Gapdh (sc-32233; Santa Cruz Biotechnology, Dallas, TX, USA), anti-TSG101 (sc-7964; Santa Cruz Biotechnology), anti-Alix (sc-53538; Santa Cruz Biotechnology), anti-HSC70 (sc-7298, Santa Cruz Biotechnology)and anti-GM130 (#610822; BD Biosciences, Tokyo, Japan) antibodies. Following incubation with alkaline phosphatase-conjugated secondary antibodies, protein signals were detected using Western Blue Stabilized Substrate.

### 2.5. Metastasis Assay

Metastasis assays were performed according to previously described procedures [15]. Briefly, mice received intravenous administration of EVs (5 µg in 100 µL PBS) or PBS alone (100 µL; control) via the tail vein at designated time points. Subsequently, *Nanog*^+^F10 cells (2.5 × 10^5^ cells in 250 µL PBS) were intravenously injected. Two weeks after tumor cell injection, mice were euthanized, and livers were collected. Liver metastasis was evaluated by gross examination and volume measurement.

### 2.6. Combination Treatment of Different EV Populations

To evaluate the combined anti-metastatic effects of different EV populations, mice were treated with combinations of EV preparations prior to melanoma cell inoculation. iPS-EVs and *Nanog*^+^F10-EVs were mixed immediately before administration. The EVs derived from *miR-466f-3p*-overexpressing F10 cells and EVs derived from *miR-19a-3p*-overexpressing F10 cells were similarly mixed immediately before administration.

Each EV preparation was administered at the same dose as that used in the corresponding single-EV treatment group (5 μg per EV preparation per mouse). Therefore, mice in the combination groups received a total of 10 μg EVs. Liver metastasis was evaluated using the same protocol described in Section 2.5.

### 2.7. Macrophage Depletion

Macrophages were depleted through administration of clodronate-loaded liposomes (Katayama Chemical Industries, Osaka, Japan). Based on previous studies [19,20,21], the treatment regimen was established as 3.1 mg/kg/week for three consecutive weeks.

### 2.8. RT-qPCR

Total cellular RNA was extracted using ISOGEN II (Nippon Gene, Tokyo, Japan). Reverse transcription was performed using SuperScript II (Thermo Fisher Scientific, Invitrogen, Waltham, MA, USA) according to the manufacturer’s instructions. RT-qPCR analysis was conducted using the StepOnePlus Real-Time PCR System (Thermo Fisher Scientific, Applied Biosystems, Waltham, MA, USA) and TB Green Premix Ex Taq II (Takara, Kusatsu, Japan). Relative target gene expression levels were normalized to Gapdh. Total RNA derived from EVs was extracted using the miRNeasy Mini Kit, and expression levels were normalized to *miR-191-5p*. Primer sequences used in this study are listed in Supplementary Table S1.

### 2.9. Enhancement of miR-19a-3p Expression in Macrophages

Two approaches were employed to increase *miR-19a-3p* expression in macrophages. In the first approach, F10 cells were transfected with a *miR-19a-3p* expression vector to generate *miR-19a-3p*^+^F10 cells, and EVs secreted from these cells were isolated from culture supernatants. The isolated EVs were then added to J774.1 cultures for cellular uptake. This approach may introduce EV-associated components in addition to *miR-19a-3p*. In the second approach, a synthetic *miR-19a-3p* mimic was introduced into J774.1 cells using Lipofectamine.

*miR-19a-3p* mimic
Guide strand (5′–3′): UGUGCAAAUCUAUGCAAAACUGAUU
Passenger strand (5′–3′): UCAGUUUUGCAUAGAUUUGCACAUU

### 2.10. miRNA Profiling and Bioinformatics Analysis

RNA extraction from EVs and small RNA sequencing (ver. MGSR3.0_mm10) were performed by Macrogen Japan (Tokyo, Japan). Fold changes and *p*-values were calculated for each miRNA. Predicted target genes were identified using TargetScanMouse 8.0, miRmap, and miRDB. Candidate genes were selected based on the following criteria: CWCS ≤ −0.40 in TargetScanMouse, percentile score ≥90 in miRmap, and target score ≥80 in miRDB. Functional enrichment analysis of selected target genes was performed using Metascape [22].

### 2.11. Statistical Analysis

Detailed statistical procedures are described in the figure legends. All experiments were independently performed at least three times as biological replicates. Data are presented as mean ± SD or mean ± SEM. Results of metastasis colony quantification were visualized as scatter plots showing all data points. Outliers were defined using the Smirnov–Grubbs test with a one-sided *p*-value threshold of 0.05 and marked as open circles. Statistical significance of the difference between two groups with unequal sample sizes was determined using a two-tailed Student’s *t*-test based on the pooled-variance of both groups. Statistical significance is indicated as follows: *** *p* < 0.001, ** *p* < 0.01, * *p* < 0.05, and † *p* < 0.1.

## 3. Results

### 3.1. Characterization of Nanog^+^F10-Derived EVs

**Figure 1:**
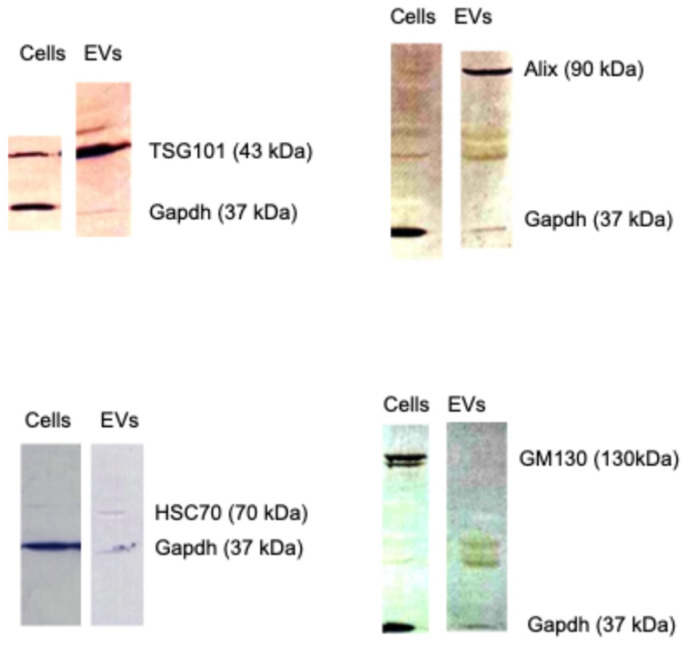
Western blotting of EV markers. The presence of TSG101, Alix and HSC70, as well as the absence of GM130, were confirmed (The original WB figure can be found in Figure S1).

*Nanog*^+^F10-EVs were isolated from culture supernatants by ultracentrifugation. Western blot analysis confirmed the presence of the EV markers TSG101, Alix and HSC70, whereas GM130, a Golgi marker used as a negative control, was not detected (Figure 1). These results indicate that the isolated EVs contained minimal contamination from intracellular components.

### 3.2. Contributions of Adaptive Immunity and Macrophages to the Anti-Metastatic Effects of Nanog^+^F10-EVs

**Figure 2:**
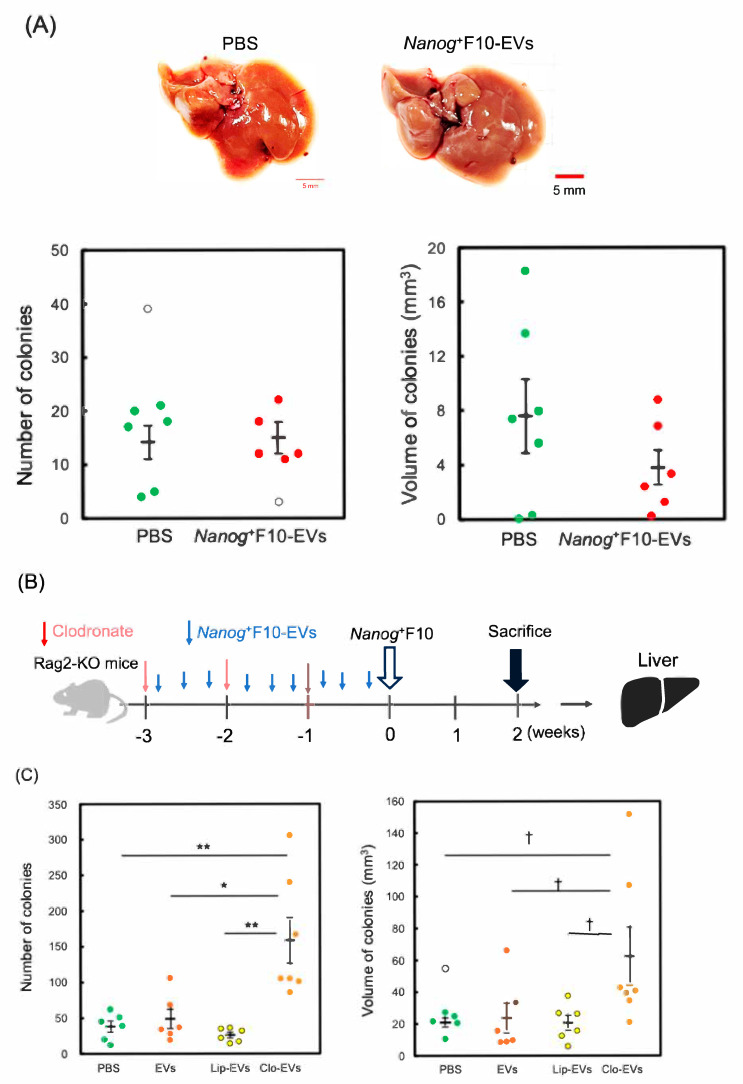
Involvement of adaptive immunity and macrophages. (**A**) Effects of Rag2−KO on the anti-metastatic effect of *Nanog*^+^F10−EVs. PBS was administered as control. PBS (*n* = 7), *Nanog*^+^F10−EVs (*n* = 6), (◦): outlier, Error bars indicate mean ± SEM. (**B**) Schedule of the administration of *Nanog*^+^F10−EVs and clodronate, the injection of *Nanog*^+^F10 cells, and the removal of liver. (**C**) Effects of clodronate on the anti-metastatic effect of *Nanog*^+^F10−EVs in Rag2−KO mice. PBS: control, EVs: *Nanog*^+^F10−EVs, Lip−EVs: Liposomes and *Nanog*^+^F10−EVs, Clo−EVs: *Nanog*^+^F10−EVs and liposomes containing clodronate. PBS (*n* = 6), EVs (*n* = 6), Lip−EVs (*n* = 6), Clo−EVs (*n* = 7), (◦) outlier, Error bars indicate mean ± SEM. ** *p* < 0.01, * *p* < 0.05 and † *p* < 0.1.

To determine whether adaptive immunity contributes to the anti-metastatic effects of *Nanog*^+^F10-EVs, liver metastasis assays were performed using Rag2-deficient mice. Under adaptive immunity-deficient conditions, metastatic burden did not differ markedly between the EV-treated and control groups (Figure 2A), suggesting that adaptive immunity makes only a limited contribution to the anti-metastatic activity of *Nanog*^+^F10-EVs. To further evaluate the role of macrophages, clodronate liposomes were administered to deplete macrophages in Rag2-deficient mice (Figure 2B). Under macrophage-depleted conditions, metastatic burden was substantially increased compared with that under control conditions (Figure 2C). These findings suggest that macrophages contribute substantially to the anti-metastatic activity of *Nanog*^+^F10-EVs.

### 3.3. Identification of Candidate miRNAs Associated with Anti-Metastatic Activity

Differential miRNA expression analysis was conducted to compare metastasis-promoting F10-EVs and metastasis-suppressing *Nanog*^+^F10-EVs. Four miRNAs were significantly upregulated (fold change >2, *p* < 0.05), whereas six miRNAs were significantly downregulated (fold change <0.5, *p* < 0.05). From these groups, the four miRNAs with the largest expression changes were selected for further analysis.

Target prediction analysis identified 405 genes that may be regulated by these eight miRNAs. Functional network analysis of the top 30 highly interconnected genes showed that *miR-19a-3p* was predicted to regulate the largest number of genes within this network (11 of 30), identifying *miR-19a-3p* as a candidate molecule associated with anti-metastatic activity.

### 3.4. Characterization of miR-19a-3p-Overexpressing Cells

**Figure 3:**
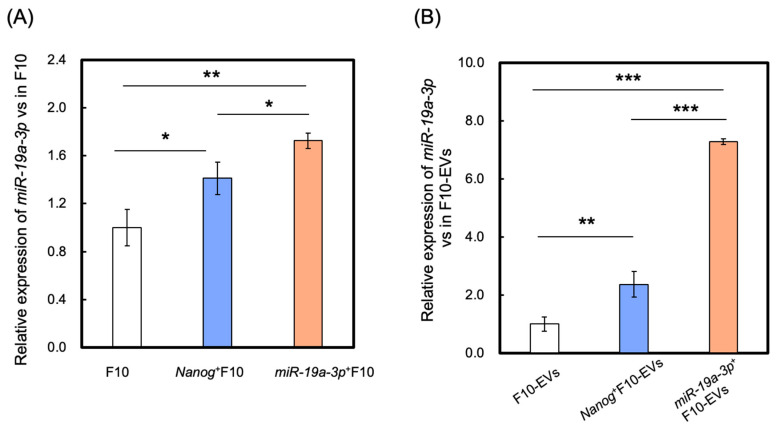

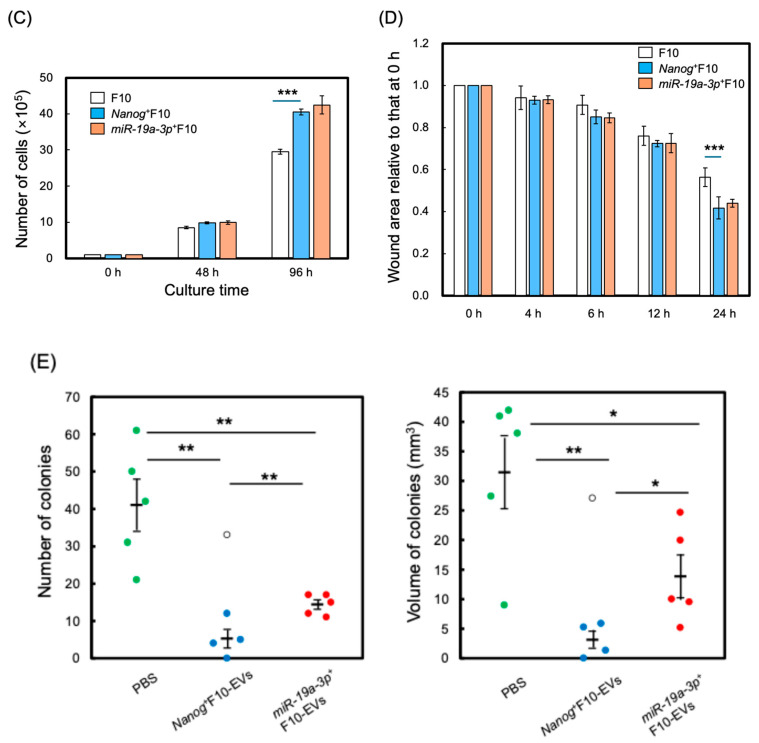
Functional properties of *miR-19a-3p*. (**A**) Expression level of *miR-19a-3p* in *miR-19a-3p*^+^F10 cells determined by qPCR. Bars indicate mean ± SD for *n* = 3. ** *p* < 0.01, * *p* < 0.05. (**B**) Expression level of *miR-19a-3p* in EVs. Bars indicate mean ± SD for *n* = 3. *** *p* < 0.001, ** *p* < 0.01. (**C**) Proliferation rate of *miR-19a-3p*^+^F10 cells as compared to F10 and *Nanog*^+^F10 cells. Bars indicate mean ± SD for *n* = 3. *** *p* < 0.001, (**D**) Migration activity of *miR-19a-3p*^+^F10 cells as compared to F10 and *Nanog*^+^F10 cells. Bars indicate mean ± SD for *n* = 3. *** *p* < 0.001. (**E**) Anti-metastatic effect of *miR-19a-3p*^+^F10−EVs as compared to *Nanog*^+^F10−EVs. PBS: control. PBS (*n* = 5), *Nanog*^+^F10−EVs (*n* = 5), *miR-19a-3p*^+^F10−EVs (*n* = 5), (◦) outlier, Error bars indicate mean ± SEM. ** *p* < 0.01, * *p* < 0.05.

To investigate the functional role of *miR-19a-3p*, we established F10 cells overexpressing *miR-19a-3p* and collected EVs secreted from these cells (*miR-19a-3p*^+^F10-EVs). *miR-19a-3p* expression levels increased stepwise in both cells and EVs across the F10, *Nanog*^+^F10, and *miR-19a-3p*^+^F10 groups (Figure 3A,B).

The biological characteristics of *miR-19a-3p*^+^F10 cells were compared with those of parental F10 and *Nanog*^+^F10 cells. Cell proliferation increased in the order of F10, *Nanog*^+^F10, and *miR-19a-3p*^+^F10 cells, although the difference between *Nanog*^+^F10 and *miR-19a-3p*^+^F10 cells was relatively modest (Figure 3C). The migratory capacity of *miR-19a-3p*^+^F10 cells was greater than that of parental F10 cells and comparable to that of *Nanog*^+^F10 cells (Figure 3D).

### 3.5. Anti-Metastatic Activity of EVs Enriched with miR-19a-3p

The anti-metastatic effects of *miR-19a-3p*^+^F10-EVs were evaluated in vivo and compared with those of PBS and *Nanog*^+^F10-EVs. Although the anti-metastatic effect of *miR-19a-3p*^+^F10-EVs was weaker than that of *Nanog*^+^F10-EVs, treatment with *miR-19a-3p*^+^F10-EVs significantly reduced metastatic burden compared with PBS, indicating that *miR-19a-3p* partially reproduced the anti-metastatic phenotype of *Nanog*^+^F10-EVs (Figure 3E).

### 3.6. Effects of miR-19a-3p on Macrophage Marker Expression

**Figure 4:**
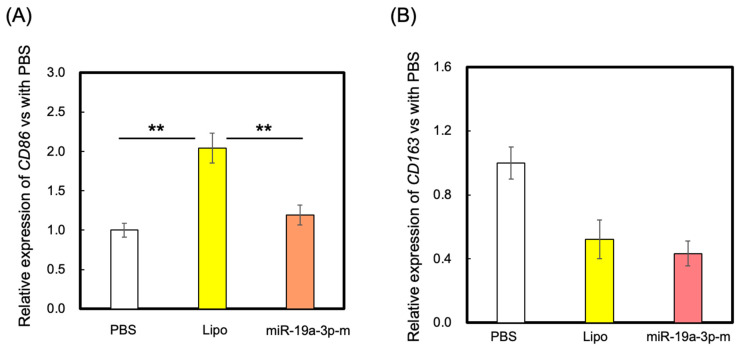
Expression of macrophage markers. (**A**) *CD86*, (**B**) *CD163*. PBS, Lipo (control): lipofectamine, *miR-19a-3p*-m: a mimic of *miR-19a-3p* added to J774.1 cells with lipofectamine. Bars indicate mean ± SD for *n* = 3. ** *p* < 0.01.

To examine the effects of *miR-19a-3p* on macrophage phenotype-associated markers, J774.1 macrophages were transfected with a *miR-19a-3p* mimic, and *CD86* and *CD163* expression levels were analyzed. *CD86* expression was significantly lower in *miR-19a-3p* mimic-transfected cells than in control cells (Figure 4A), whereas *CD163* expression showed no significant change (Figure 4B).

### 3.7. Bioinformatics Analysis of Candidate Signaling Pathways

**Figure 5:**
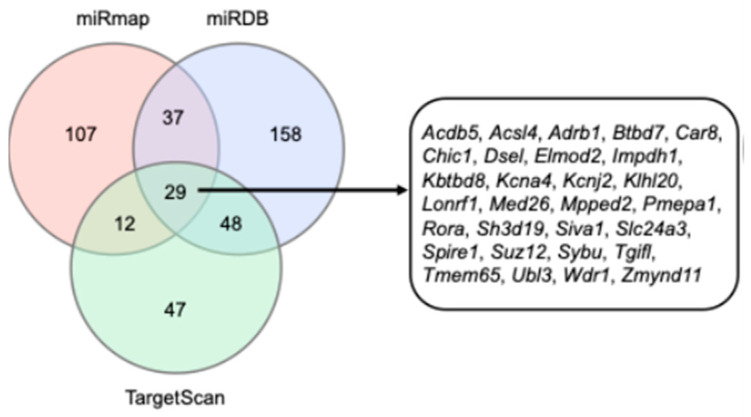
Predominant target genes of *miR-19a-3p* predicted by three databases. Twenty-nine genes were nominated.

Target prediction analysis of *miR-19a-3p* using TargetScan, miRDB, and miRmap identified 29 overlapping genes (Figure 5). Among these genes, STRING functional annotation analysis identified five genes associated with immune-related functions. Metascape pathway analysis showed that the most significantly enriched pathway cluster was related to NF-κB-associated signaling pathways (Table 1). This cluster included *Rora*, *Siva1*, and *Zmynd11*, suggesting that these molecules may be involved in *miR-19a-3p*-mediated signaling pathways.

### 3.8. Enhanced Anti-Metastatic Effects of Combined EV Populations

To determine whether combined administration of different EV populations enhanced anti-metastatic activity, mice were treated with mixtures of EVs prior to melanoma cell inoculation.

**Figure 6:**
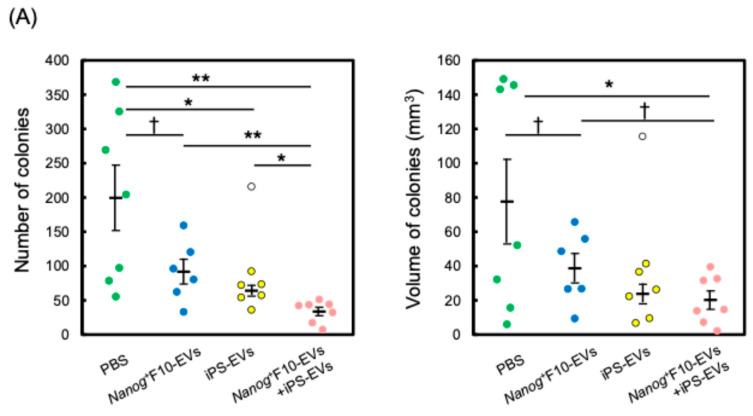

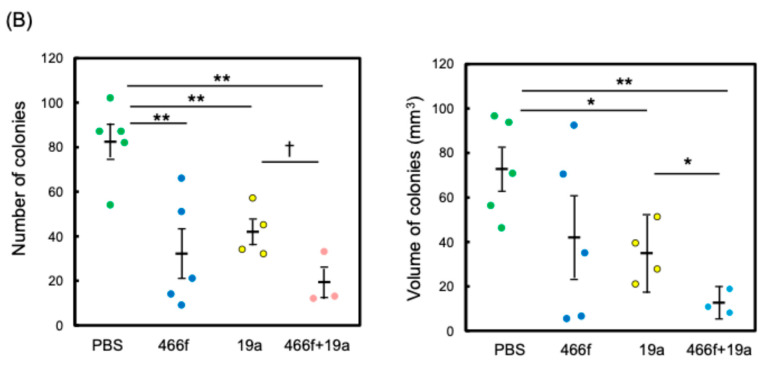
Enhanced anti-metastatic effects of combined EVs treatment. (**A**) Combined administration of *Nanog*^+^F10−EVs and iPS−EVs in the melanoma liver metastasis model. Mice received PBS, *Nanog*^+^F10−EVs alone, iPS−EVs alone, or a combination of *Nanog*^+^F10−EVs and iPS−EVs. For the combination group, each EV preparation was administered at the same dose as that used in the corresponding single-EV group (5 μg each; total 10 μg per mouse). Numbers in parentheses indicate the number of mice in each group: PBS (*n* = 7), *Nanog*^+^F10−EVs (*n* = 6), iPS−EVs (*n* = 7), and *Nanog*^+^F10−EVs + iPS−EVs (*n* = 7). (◦) outlier, ** *p* < 0.01, * *p* < 0.05, † < 0.1. (**B**) Combined administration of EVs derived from *miR-466f-3p*^+^F10 cells and *miR-19a-3p*^+^F10 cells in the melanoma liver metastasis model. Mice received PBS, *miR-466f-3p*^+^F10−EVs (466f), *miR-19a-3p*^+^F10−EVs (19a), or a combination of both EV preparations (466f + 19a). Each EV preparation was administered at the same dose as that used in the corresponding single-EV group (5 μg each; total 10 μg per mouse). Numbers in parentheses indicate the number of mice in each group: PBS (*n* = 5), 466f (*n* = 5), 19a (*n* = 4), and 466f + 19a (*n* = 3). (◦) outlier, Error bars indicate mean ± SEM. ** *p* < 0.01, * *p* < 0.05, † *p* < 0.1.

Co-administration of iPS-EVs and *Nanog*^+^F10-EVs significantly reduced the number of metastatic liver nodules compared with treatment using either EV preparation alone (Figure 6A). Under the experimental conditions used in this study, combined EV treatment exhibited greater anti-metastatic activity than either individual EV preparation.

We next examined whether similar effects were observed using EVs enriched with different functional miRNAs. Co-administration of *miR-466f-3p*-enriched EVs and *miR-19a-3p*-enriched EVs also resulted in greater suppression of liver metastasis than either EV preparation alone (Figure 6B).

These findings demonstrate that combined administration of distinct EV populations enhanced anti-metastatic activity in vivo. The mechanisms responsible for this enhanced activity remain to be determined.

## 4. Discussion

In this study, we investigated the mechanisms by which *Nanog*^+^F10 cell-derived EVs suppress melanoma metastasis using a *Nanog*^+^F10 liver metastasis model. Our findings suggest that the anti-metastatic effects of *Nanog*^+^F10-EVs appears to be mediated primarily by innate immunity, particularly macrophages, rather than by adaptive immunity. The observation that the anti-metastatic effect was not substantially reduced in Rag2-deficient mice, whereas metastasis increased following macrophage depletion, is consistent with the possibility that this effect is exerted mainly through the innate immune system. However, because a clodronate-only control group was not included, the independent effect of macrophage depletion on metastatic burden cannot be completely excluded. Therefore, although our findings support a major contribution of macrophages to the anti-metastatic activity of *Nanog*^+^F10-EVs, additional experiments including appropriate depletion controls will be required to further strengthen this conclusion.

miRNA profiling revealed that *miR-19a-3p* was highly enriched in *Nanog*^+^F10-EVs. In addition, EVs derived from *miR-19a-3p*-overexpressing cells partially reproduced the anti-metastatic effects of *Nanog*^+^F10-EVs, suggesting that this miRNA may be a candidate functional mediator. However, expression of *miR-19a-3p* in J774.1 cells significantly reduced the expression of *CD86*, an M1 marker, while causing no apparent change in *CD163*, an M2 marker. These results suggest that *miR-19a-3p* does not simply drive macrophages toward either an M1-like or M2-like phenotype. Recent studies have shown that macrophage functional states cannot be fully explained by the conventional M1/M2 dichotomy and include diverse intermediate and stimulus-dependent states [3,23,24]. Thus, the anti-metastatic effects of *Nanog*^+^F10-EVs may not result from typical M1-like polarization. Instead, our findings suggest that EVs containing *miR-19a-3p* may regulate macrophage functional states in a manner that cannot be adequately described by the M1/M2 dichotomy.

EVs carry diverse molecular cargo, including miRNAs, proteins, and lipids, and mediate intercellular communication [4,25]. Recent studies have also reported that EVs can alter immune-cell functional states rather than acting solely as molecular carriers [9,26]. This is consistent with the possibility that *miR-19a-3p*-associated EVs contributed to metastasis suppression through innate immune regulation in the present study. Previous studies have shown that MSC-derived EVs mainly induce M2 macrophages and exert immunosuppressive effects [27,28], whereas EVs derived from cells with stem cell-like properties can regulate metastasis and the tumor microenvironment [13,29]. More recent evidence also suggests that some EVs may influence immune responses by inducing inflammatory macrophages [9]. In this context, our findings support the concept that the immune regulatory functions of EVs vary according to the state of their cells of origin.

In contrast, *miR-19a-3p* promoted tumor cell proliferation and migration. *miR-19a-3p* is a representative oncomiR of the *miR-17-92* cluster and has been implicated in cell proliferation, migration, and tumor progression [30,31,32]. Consistent with previous reports, tumor cells overexpressing *miR-19a-3p* in this study exhibited increased proliferative and migratory abilities.

Although these findings may appear inconsistent with the observed anti-metastatic effects, our data suggest that EV-mediated effects are exerted mainly through immune cells. Thus, *miR-19a-3p* may exert distinct functions depending on the target cell type. Recent studies have reported that miRNAs derived from the *miR-17-92* cluster, including *miR-19a-3p*, participate in the regulation of immune-cell functions and inflammatory responses [33,34]. Accordingly, *miR-19a-3p* may promote tumor-associated phenotypes in tumor cells while altering immune-cell functional states in a manner not reducible to M1/M2 polarization, thereby contributing to anti-metastatic innate immune responses.

This dual role suggests that the functions of EV-mediated miRNAs are strongly influenced by the target cell type and intracellular regulatory networks. The present findings indicate that *miR-19a-3p* may function not only as a tumor-promoting factor but also as a regulator of innate immune-cell functional states when delivered through EVs.

Although EVs enriched in *miR-19a-3p* partially reproduced the anti-metastatic activity of *Nanog*^+^F10-EVs, the present study relied primarily on gain-of-function approaches. Therefore, it remains unclear whether *miR-19a-3p* is required for the anti-metastatic activity of *Nanog*^+^F10-EVs. Future loss-of-function studies will be necessary to determine the extent to which *miR-19a-3p* contributes to this biological effect and whether additional EV-associated molecules also participate in the observed anti-metastatic activity. Accordingly, the present findings support the interpretation that *miR-19a-3p* is a candidate functional mediator of the anti-metastatic activity of *Nanog*^+^F10-EVs, rather than establishing it as the sole determinant of this biological effect.

To explore potential downstream mechanisms underlying the effects of *miR-19a-3p*, bioinformatics analysis identified several candidate target molecules, although their detailed molecular mechanisms require further investigation. Because *miR-19a-3p* can regulate multiple target molecules, its effects may be mediated through several signaling pathways rather than a single target [35,36]. In this study, *Siva1*, *Zmynd11*, and *Rora* were predicted as candidate targets of *miR-19a-3p*. These molecules have been associated with cellular stress responses, transcriptional regulation, and immune homeostasis [37,38,39,40]. *Siva1* has been linked to apoptosis and cellular stress responses [37], *Zmynd11* is known to regulate transcription through histone modification [38], and *Rora* has been reported to contribute to immune homeostasis and macrophage functional regulation [39,40]. These findings suggest that *miR-19a-3p* may regulate immune-cell function through network-level control involving multiple signaling pathways rather than through a single target molecule. However, the direct regulation of these candidate molecules was not experimentally validated in this study, and further analyses are needed to define the molecular basis of this mechanism.

The present findings suggest that EV-derived miRNAs may alter the functional states of immune cells, thereby reshaping innate immune responses. These findings may share certain features with the concept of trained immunity, in which innate immune cells acquire altered responsiveness following prior stimulation [41,42,43]. In particular, the finding that EVs administered before metastasis affected subsequent metastatic formation suggests that EVs may regulate future immune responsiveness in advance.

Furthermore, co-administration of iPS-EVs and *Nanog*^+^F10-EVs produced greater suppression of liver metastasis than either EV preparation alone (Figure 6A). A similar enhancement was observed when EVs enriched in *miR-466f-3p* were combined with EVs enriched in *miR-19a-3p* (Figure 6B). These findings indicate that combined administration of distinct EV populations enhanced anti-metastatic activity under the experimental conditions used in this study. However, because each EV preparation was administered at the same dose as that used in the corresponding single-EV treatment group, the combination groups received a greater total amount of EVs (10 μg versus 5 μg in the individual treatment groups). Therefore, the contribution of increased EV dose to the enhanced anti-metastatic activity cannot be completely excluded. Further dose-matched studies will be required to distinguish between quantitative and qualitative contributions to the observed enhancement. Nevertheless, the observation that similar enhancement was obtained using both EVs derived from different cellular origins and EVs carrying different functional miRNAs suggests that qualitative differences in EV cargo may also contribute to the enhanced biological activity.

Nevertheless, these observations raise the possibility that EVs should not be viewed simply as individual carriers of bioactive molecules but rather as what we term “cooperative information units”, in which distinct EV populations carrying different molecular cargoes act in a complementary manner to regulate innate immune-cell function. In this model, each EV population may contribute only part of the overall biological response, whereas coordinated actions of multiple EV populations may generate a more robust anti-metastatic immune state than can be achieved by individual EV populations alone.

Based on these observations, our findings further suggest a model of preemptive immune education, in which EVs delivered before metastatic challenge may condition innate immune cells into a functional state that enhances subsequent anti-tumor responses. Rather than directly eliminating tumor cells, EVs may establish an immune environment that is more responsive to future metastatic insults. This concept extends the conventional view of EVs as molecular delivery vehicles by proposing that they may also function as biological educators that shape future immune responsiveness. Although this model requires further experimental validation, it provides a conceptual framework for understanding how multiple EV populations may cooperatively regulate innate immunity and may facilitate the development of next-generation EV-based strategies for metastasis prevention.

EVs have attracted attention as next-generation molecular delivery and therapeutic platforms because of their low immunogenicity and high molecular delivery capacity [44,45,46]. Although EV research has traditionally focused mainly on their role as molecular transporters, the present findings suggest that EVs may also function as information platforms capable of directing immune responses. In particular, the anti-metastatic effects observed in this study appear to be mediated primarily through macrophage-dependent innate immune mechanisms rather than through direct effects on tumor cells. This suggests that EV-derived miRNAs may preemptively educate immune cells and alter their future responsiveness to the tumor microenvironment. Moreover, the enhanced anti-metastatic effects observed after combining different EVs suggest that EVs may cooperatively direct immune states as multiple information units rather than acting independently.

Taken together, the present findings suggest that *Nanog*^+^F10-EVs suppress melanoma metastasis primarily through modulation of innate immune-cell functional states, particularly those of macrophages. Although the precise molecular mechanisms remain to be fully elucidated, our data are consistent with a conceptual model of preemptive immune education, in which EVs delivered before metastatic challenge may condition innate immune cells into a functional state that enhances subsequent anti-metastatic responses. While this concept requires further experimental validation, it provides a useful framework for understanding EV-mediated innate immune regulation and may contribute to the future development of EV-based immunotherapeutic strategies for metastasis prevention.

## 5. Conclusions

In conclusion, the present study demonstrates that extracellular vesicles derived from *Nanog*-overexpressing melanoma cells suppress experimental melanoma metastasis predominantly through macrophage-dependent innate immune mechanisms rather than through direct effects on tumor cells. Our findings further identify *miR-19a-3p* as a candidate functional mediator associated with this biological activity, although additional loss-of-function studies will be required to establish its causal contribution. These results also suggest that the anti-metastatic effects of *Nanog*^+^F10-EVs cannot be fully explained by conventional M1/M2 macrophage polarization, supporting the concept that EVs modulate innate immune-cell functional states in a more complex manner.

Furthermore, the enhanced anti-metastatic activity observed following the combined administration of distinct EV populations suggests that different EV populations may function cooperatively through complementary molecular cargoes. We therefore propose that EVs can be regarded as *cooperative information units* that collectively mediate *preemptive immune education*, whereby EVs delivered before metastatic challenge condition innate immune cells into a functional state that enhances subsequent anti-tumor responses. Although further mechanistic studies are required, this conceptual framework provides new insight into EV-mediated immune regulation and may facilitate the development of next-generation EV-based strategies for the prevention of cancer metastasis.

## Figures and Tables

**Table 1:** Predominant pathways predicted by enrichment analysis using Metascape.

Examples of Pathway in the Cluster			
Description	Enrichment	Log (*p*)	Hit Genes
Negative regulation of canonical NF-kappaB signal transduction	22.1	−3.5	*Rora*, *Siva1*, *Zmynd11*
Regulation of canonical NF-kappaB signal transduction	7.9	−2.2	*Rora*, *Siva1*, *Zmynd11*
Potassium ion transmembrane transport	13.5	−2.9	*Kcna4*, *Kcnj2*, *Slc24a3*
Potassium ion transport	12.0	−2.7	*Kcna4*, *Kcnj2*, *Slc24a3*
Regulation of membrane potential	5.7	−2.3	*Adrb1*, *Kcna4*, *Kcnj2*, *Wdr1*
Regulation of heart contraction	11.0	−2.6	*Adrb1*, *Kcnj2*, *Tmem65*
Regulation of blood circulation	8.1	−2.2	*Adrb1*, *Kcnj2*, *Tmem65*
Antigen processing: ubiquitination and proteasome degradation	8.3	−2.3	*Klhl20*, *Kbtbd8*, *Lonrf1*
Class I MHC mediated antigen processing and presentation	6.8	−2.0	*Klhl20*, *Kbtbd8*, *Lonrf1*

## Data Availability

The original contributions presented in this study are included in the article. Further inquiries can be directed to the corresponding author.

## Supplementary Materials

The following supporting information can be downloaded at: https://www.mdpi.com/article/10.3390/cancers18142200/s1. Figure S1: Western blot analysis of EV markers; Table S1: Primers for qPCR.
