# Supplementary material for: Nanog+F10-Derived Extracellular Vesicles Suppress Melanoma Metastasis, Implicating miR-19a-3p in Macrophage-Dependent Innate Immune Regulation
Source: Cancers (Basel). 2026 Jul 8;18(14):2200. doi: 10.3390/cancers18142200 (PMC13406691; doi:10.3390/cancers18142200)
Supplement: Supplementary file 1 [file cancers-18-02200-s001.zip › cancers-4354452-supplementary Figure S1.pdf]

Fig. S1. Western blot analysis of EV markers.

The intensity ratio was calculated using the formula:

EV[target band/GAPDH band]/Cells[target band/GAPDH band].

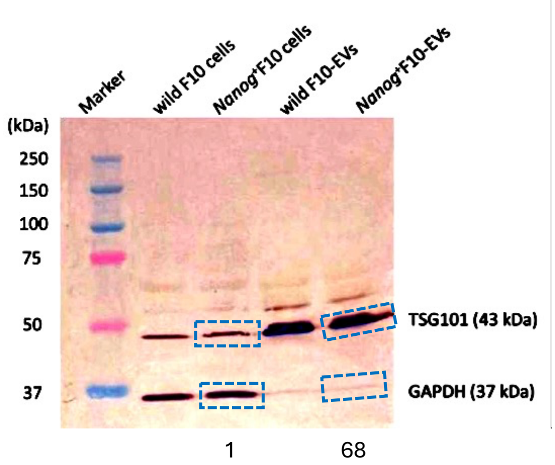

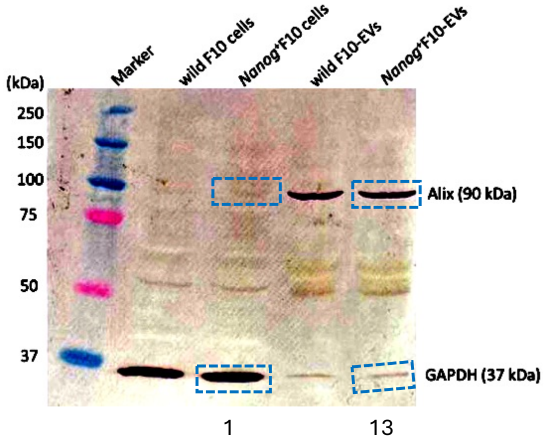

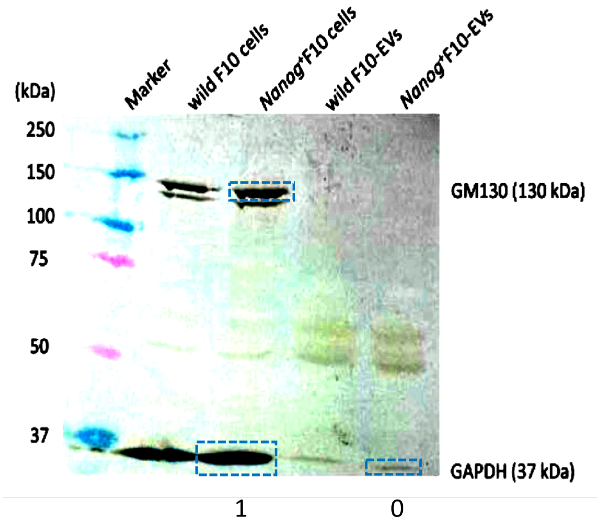

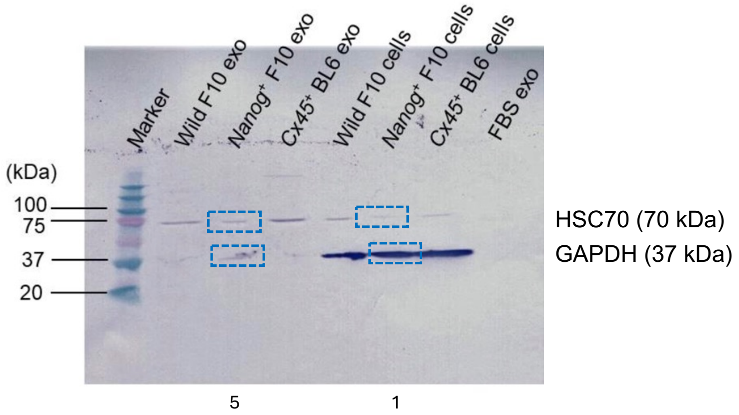
Marker
Wild F10 exo
Nanog+ F10 exo
Cx45+ BL6 exo
Wild F10 cells
Nanog+ F10 cells
Cx45+ BL6 cells
FBS exo
(kDa)
100
75
37
20
HSC70 (70 kDa)
GAPDH (37 kDa)
5
1
