# Supplementary material for: Nanog+F10-Derived Extracellular Vesicles Suppress Melanoma Metastasis, Implicating miR-19a-3p in Macrophage-Dependent Innate Immune Regulation
Source: Cancers (Basel). 2026 Jul 8;18(14):2200. doi: 10.3390/cancers18142200 (PMC13406691; doi:10.3390/cancers18142200)
Supplement: Supplementary file 1 [file cancers-18-02200-s001.zip › cancers-4354452-supplementary Table S1.pdf]

Table S1. Primers for qPCR

	Forward primer	Reverse primer
miR-19a-3p	CACGCATGTGCAAATCTATGCA	CCAGTGCAGGGTCCGAGGTA
miR-191-5p	CGCACAACGGAATCCCAAAAG	CCAGTGCAGGGTCCGAGGTA
CD86	CATGGGCTTGGCAATCCTTA	AAATGGGCACGGCAGATATG
CD163	GGCTAGACGAAGTCATCTGCAC	CTTCGTTGGTCAGCCTCAGAGA
GAPDH	TGTGTCCGTCGTGGATCTGA	CCTGCTTCACCACCTTCTTGA
